# Investigation of the Cytotoxic and Antiproliferative Effects of Liposomal Daunorubicin on Human Colorectal Cancer (HCT116) Cell Line

**DOI:** 10.5812/ijpr-144287

**Published:** 2024-05-29

**Authors:** Noorulhuda Alaa Hadi-al-Ward, Mehdi Ebrahimi, Shohre Zare Karizi

**Affiliations:** 1 Department of Genetics, Faculty of Converging Sciences and Technologies, Science and Research Branch, Islamic Azad University, Tehran, Iran; 2 Department of Biochemistry and Biophysics, College of Biological Sciences, Varamin-Pishva branch, Islamic Azad University, Pishva, Iran; 3 Department of Genetics, Faculty of Biological Sciences, Varamin-Pishva branch, Islamic Azad University, Pishva, Iran

**Keywords:** Colorectal Cancer, Daunorubicin, Liposomes, Phosphatidyl-Inositol 3-Kinase, Cell Cycle, HCT116 Cells

## Abstract

**Background:**

The incidence of colorectal cancer is increasing globally. Daunorubicin (DNR), an anthracycline antibiotic, is effective against various cancers. The PI3K/AKT/mTOR signaling pathway is crucial in regulating cell growth and cancer growth.

**Objectives:**

This study aims to evaluate the effects of liposomal daunorubicin (Lip-DNR) on cell proliferation and cell death induction in HCT116 cells compared to free daunorubicin.

**Methods:**

Lip-DNR was synthesized, and its shape and size were analyzed using FE-SEM imaging. HCT116 cells were treated with Lip-DNR concentrations of 0 (control), 0.125, 0.25, 0.5, 1, and 2 μm for 48 hours to determine the IC50. The effects of free (0.5 μm) and liposomal DNR (IC50 of 0.43 μm) on PI3K mRNA levels were assessed using real-time PCR. The cell cycle was analyzed by flow cytometry.

**Results:**

FE-SEM imaging showed that the liposomes are spherical and range from 50 - 100 nm in size. Lip-DNR induced cell death in HCT116 cells in a dose-dependent manner, with 0.5 μm Lip-DNR causing more cell death than an equivalent concentration of free DNR. Analysis of PI3K gene expression showed that DNR decreases PI3K gene transcription in HCT116 cells, with Lip-DNR having a more substantial effect than the free form. Both forms reduced the proportion of G2/M phase cells, but Lip-DNR was more effective at inhibiting cell proliferation in HCT116 cells.

**Conclusions:**

DNR inhibits the proliferation of HCT116 cells by downregulating PI3K gene expression and enhancing cell death, with the liposomal form demonstrating stronger effects than the free form.

## 1. Background

Colorectal cancer (CRC) arises from the uncontrolled proliferation of glandular epithelial cells in the colon or rectum. The incidence of CRC is increasing daily (1). It ranks as the third most common cancer in men and the second in women globally. In 2020, over 1.9 million new cases were reported, and CRC was the second leading cause of cancer death, responsible for approximately 935 000 deaths (2). Globally, it accounts for 11% of all cancer diagnoses and is one of the cancers with a rising incidence rate (3). To manage tumors, chemotherapy or radiation therapy is often applied before or after surgery, but these treatments come with severe side effects like systemic toxicity, low response rates, resistance to chemotherapy agents, and poor tumor selectivity, prompting ongoing research to develop and refine chemotherapeutic approaches (4).

Anthracyclines, a class of natural antibiotics, are considered among the most effective chemotherapeutic agents used clinically. The first compounds, daunorubicin (DNR) and doxorubicin, were introduced for clinical trials (5). However, DNR use can lead to significant adverse effects, including tumor cell resistance, damage to healthy tissues, and notably heart failure characterized by chronic cardiomyopathy and congestion (6). Liposomes are frequently employed to mitigate these side effects and enhance biomedical treatments by stabilizing therapeutic compounds, increasing cellular and tissue uptake, and enhancing drug delivery to target sites (7). Composed of phospholipids with a polar phosphate head and hydrophobic lipid tails, liposomes form a globular structure in aqueous environments, encapsulating an aqueous core within a lipophilic bilayer membrane (8). These features render liposomes effective nanocarriers for targeted drug delivery (7) due to their biocompatibility, ability to transport large drug payloads, and adjustable physical, chemical, and biophysical properties to control drug behavior (9).

Despite its benefits, the exact mechanism underlying the antineoplastic effects of daunorubicin (DNR) remains unclear. The PI3K/mTOR/AKT pathway is crucial for regulating cell growth, motility, survival, metabolism, and angiogenesis, and is found to be dysregulated in nearly all types of human cancers, including breast cancer, CRC, and hematological malignancies. This underscores the importance of targeting this pathway as a potential therapeutic strategy in cancer treatment (10, 11). Phosphoinositide 3-kinases (PI3Ks) specifically add a phosphate group to the D3 position of the inositol ring in membrane phosphoinositides (PI). Class I PI3Ks can produce phosphatidylinositol 3,4-bisphosphate and phosphatidylinositol 3,4,5-triphosphate, which, although present in very low concentrations in stationary G0 cells, increase rapidly and transiently following growth factor receptor stimulation, significantly influencing cell survival, migration, and division (11).

The quest for more effective CRC treatments has led to the exploration of new drugs as a promising research area. While DNR has been established as an effective treatment for acute myeloid leukemia (AML), its potential efficacy for treating CRC has yet to be assessed. 

## 2. Objectives

This study aims to investigate the impact of liposomal DNR on the proliferation and cell death of HCT116 colon cancer cells.

## 3. Methods

### 3.1. Preparation of Liposomal Daunorubicin

Liposomes containing DNR were prepared using the thin-layer hydration method, described by Y. Chen et al. in 2012. Initially, a mixture of DNR (1 μm), lecithin (40 mg), and cholesterol (5 mg) was dissolved in 10 mL of chloroform. This solution was then dried using a rotary evaporator at 50°C to form a thin lipid layer. To this dried layer, saline phosphate buffer solution (pH 6.5, 1 mL) and Tween-80 (5 mg) were added. The mixture was stirred at 50°C for 30 minutes until completely dissolved (hydration stage). The resultant solution was then sonicated for 3 minutes at 80 W. The liposomes were stored at 4°C, and their shape and size were characterized using FE-SEM (Mira 3-XMU).

### 3.2. Cell Culture

HCT-116 cells (National Center for Genetic Resources of Iran) were cultured in DMEM medium supplemented with 10% fetal bovine serum at 37°C with 5% CO_2_. To detach the cells from the container's bottom, 1 mL of Trypsin-EDTA solution (0.25% trypsin and 1 mM EDTA) was applied to each well and left for 5 minutes. The trypsin activity was neutralized by adding 2 ml of culture medium containing 10% FBS to the cells, followed by centrifugation at 300 g for 5 minutes.

### 3.3. Cytotoxicity Assay

To evaluate the cytotoxic effects of various treatments, an MTT assay was conducted. The treatments included a control (untreated), Lip-DNR at concentrations of 0.125, 0.25, 0.5, 1, and 2 μm, and free DNR at 0.5 μm, each replicated three times. A 1 mM concentration of the DNR drug (Molar mass: 527.52 g/mol) was dissolved in DMSO and the liposomal drug was diluted in the culture medium to achieve the desired final concentrations. Approximately 2500 cells were cultured in DMEM medium with 10% fetal bovine serum at 37°C and 5% CO_2_ for 48 hours. Next, 100 µL of MTT solution (5 mg/mL in PBS) was added and incubated for 3 to 4 hours at 37°C. The solution was then removed, and 100 µL of DMSO was added to each well and incubated for 15 minutes at 37°C. Finally, the absorbance was measured at 570 nm. The survival percentage of each sample was calculated using the formula:


$$Survival\;percentage\;of\;each\;sample=(\frac{Absorbance\;of\;treatment}{Absorbance\;of\;control})\times 100$$

### 3.4. Investigation of PI3K Gene Transcription

To investigate the PI3K gene transcription level, the following steps were taken for total RNA extraction: After draining the supernatant from the 48-hour cultured cells, the cells were washed with PBS. The cells were lysed using 1 mL of Trizol (guanidinium thiocyanate-phenol), followed by the addition of 200 µL of chloroform for 15 minutes at room temperature. After centrifugation for 15 minutes at 4°C and 12 000 rpm, 500 µL of isopropanol was added to the supernatant (containing RNA) and incubated for 10 minutes at -20°C. The samples were then centrifuged at 12 000 rpm for 10 minutes at 4°C. The supernatant was discarded, and the RNA pellet was washed with 200 µL of 75% ethanol to remove impurities. Finally, centrifugation at 7 500 rpm for 5 minutes at 4°C was performed to remove the ethanol. The RNA pellet was resuspended in 30 µL of RNase-free water.

The Easy cDNA Synthesis Kit (Pars Tos, Iran) was utilized for cDNA synthesis according to the manufacturer's instructions. The mRNA levels of PI3K and GAPDH were quantitatively measured using the real-time PCR method. The primer sequences for the PI3K gene were 5'-GGA AAG GTG GGA GGG GAG GT-3' and 5'-TCT GAG GGT GAG GAA GGA GGT-3', and for the GAPDH gene (control) were 5'-CTT TGG TAT CGT GGA AGG AC-3' and 5'-GCA GGG ATG ATG TTC TGG-3'. The PCR reaction included 5 µL of SYBR Green Master Mix, 0.5 µL of both forward and reverse primers (10 µM), 3 µL of sterile distilled water, and 3 µL of the cDNA sample, making a total volume of 10 µL. The PCR cycle settings on the ABI StepOne real-time PCR thermocycler (USA) were: Initial denaturation at 95°C for 5 minutes, followed by 40 cycles of denaturation at 95°C for 15 seconds, annealing at 60°C for 20 seconds, and extension at 72°C for 30 seconds, with a final step at 30°C for 20 seconds. Data from the real-time PCR were used to calculate fold changes. Statistical analysis was conducted using GraphPad Prism software, employing a one-way ANOVA to evaluate differences between groups, with Tukey's post hoc test to assess the significance of these differences.

### 3.5. Cell Cycle Evaluation

Changes in the cell cycle of HCT116 cells cultured for 48 hours were investigated using flow cytometry and propidium iodide (PI) staining. Propidium iodide stains double-stranded DNA by intercalating between the DNA bases after penetrating dead cells. Propidium iodide excitation occurs at 488 nm and it emits light at 617 nm. The fluorescence levels of the samples were measured using a flow cytometer (BD Biosciences, Belgium).

### 3.6. Statistical Analysis

Statistical analyses were performed using GraphPad Prism software. ANOVA and/or *t*-tests were used to determine the significance of differences between groups. Results were expressed as mean ± standard error of the mean (SEM). A P-value of less than 0.05 was considered statistically significant. All experiments were independently performed at least three times.

## 4. Results

### 4.1. Shape and Size of Liposomes 

**Figure 1.**
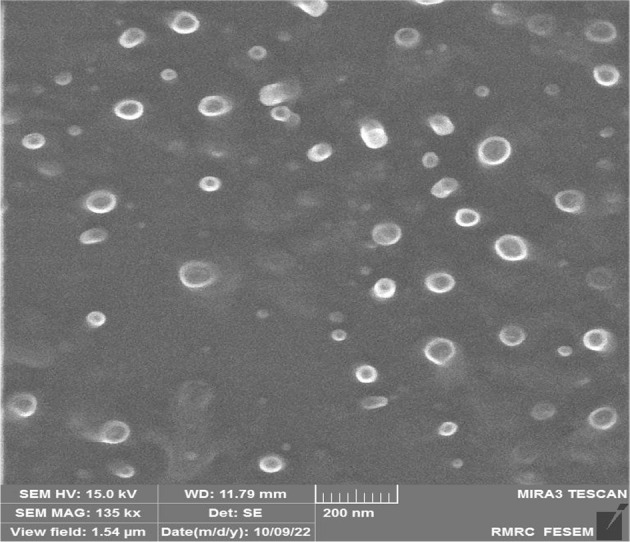
FE-SEM image displaying synthesized liposomes, which are spherical and have diameters ranging from 50 to 100 nm

Liposomes encapsulating the drug daunorubicin (DNR) were synthesized using the thin layer hydration method. The shape and size of these liposomes were analyzed using the FE-SEM method. The images revealed that the liposomes are spherical and range in size from 50 to 100 nm (Figure 1). 

### 4.2. Cell Morphology Assessment 

**Figure 2.**
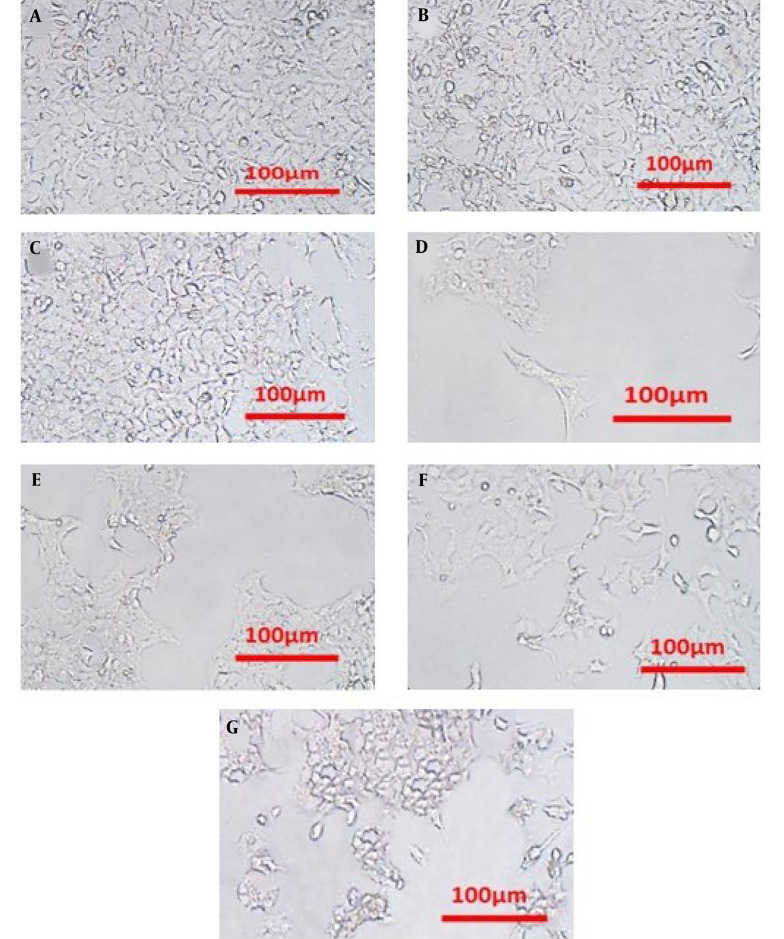
Phase contrast microscope images of HCT116 cells after 48 hours of treatment showing A, the control group; B - F, varying concentrations of Lip-DNR (0.125, 0.25, 0.5, 1, and 2 μm); and (G) DNR at 0.5 μm

Phase contrast microscopy was employed to evaluate the cytotoxic effects on HCT116 cells in three different groups: Control, DNR, and liposomal DNR (Lip-DNR) (Figure 2). The groups treated with DNR and Lip-DNR showed reduced cell density and increased numbers of spherical cells, exhibiting characteristics of apoptosis compared to the control. Additionally, the rate of cell death escalated with higher concentrations of Lip-DNR.

### 4.3. Investigation of Cytotoxicity by MTT Method and Determination of IC50 

**Figure 3.**
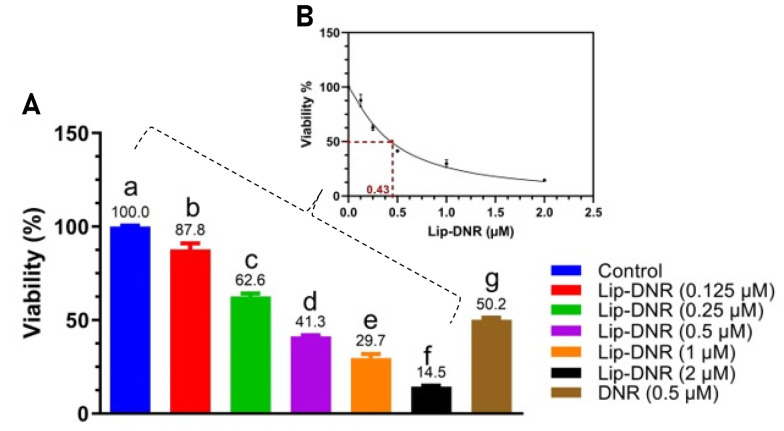
A, MTT test results for control groups, Ctrl; liposomal DNR, Lip-Dau; Daunorubicin, DNR. B, IC50 determination for Lip-DNR. Different letters above the columns indicate P < 0.05

The MTT assay was conducted to assess the cytotoxicity of both DNR and Lip-DNR. Results depicted in Figure 3A indicate a significant reduction in the viability of cells treated with DNR (0.5 μm), which was 50% lower than that of the control (P < 0.0001). Lip-DNR exhibited dose-dependent cytotoxic effects; at the same 0.5 μm concentration, Lip-DNR reduced cell viability by 8.7% more than the control (P = 0.030). At the lowest tested concentration of Lip-DNR (0.125 μm), cell death was 12.2% higher than the control (P < 0.0024), while at the highest concentration (2 μm), it was more than 85% above the control (P < 0.0001). These results suggest that Lip-DNR's cytotoxic properties are dose-dependent and more potent at inducing cell death than an equivalent concentration of free DNR. The IC50 for Lip-DNR was determined to be 0.43 μm (Figure 3B). 

### 4.4. Evaluation of Relative PI3K mRNA Level

**Figure 4.**
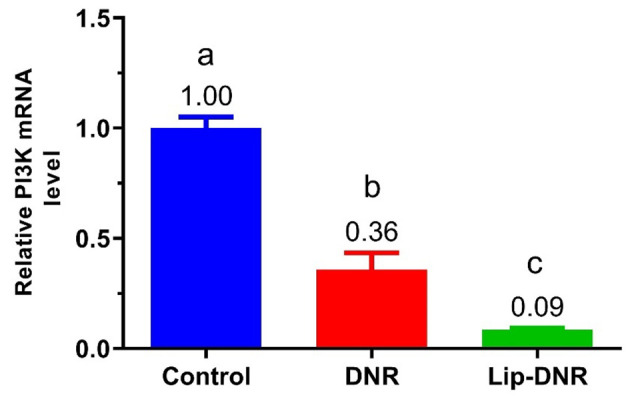
Comparison of the relative level of PI3K mRNA in cell groups: Control, Ctrl.; Daunorubicin (0.5 μm), DNR; Liposomal daunorubicin (0.43 μm), Lip-DNR. Different letters above the columns indicate P < 0.05

Figure 4 shows changes in PI3K gene expression in HCT116 cell groups. PI3K expression in the DNR (0.5 μm) and Lip-DNR (0.43 μm) groups was 0.6-fold (P = 0.0003) and 0.9-fold (P < 0.0001) lower than the control group, respectively. Additionally, PI3K gene expression in the DNR group was 0.3 times lower than in the Lip-DNR group (P = 0.024). These results demonstrate that DNR reduces PI3K gene transcription in HCT116 cells, with Lip-DNR having a more significant effect.

### 4.5. Cell Cycle Assessment

**Figure 5.**
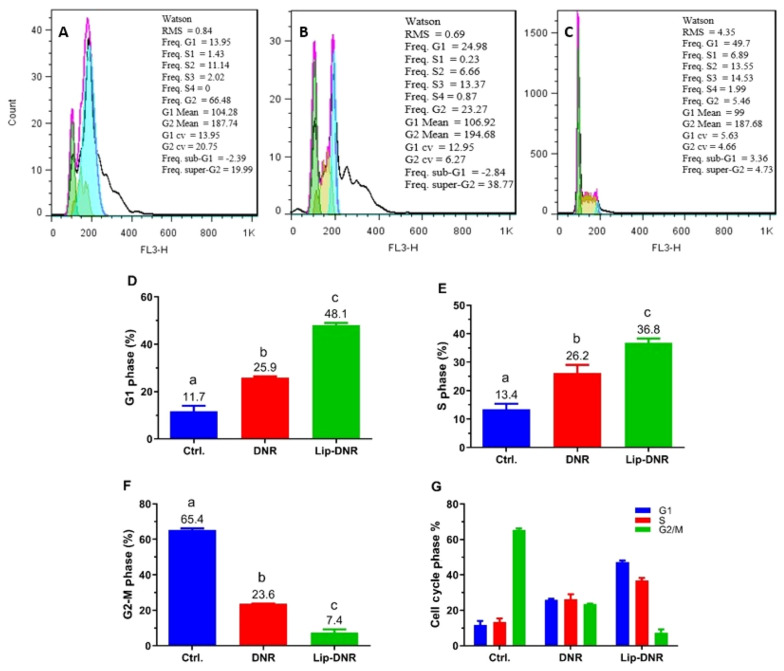
Results from the cell cycle analysis by flow cytometry. A, B, and C, Results evaluation using FlowJo software; D, E, and F, Distribution of HCT116 cells in the G1, S, and G2/M phases of the cell cycle, respectively; G, Percentage distribution of cells in each group across G1, S, and G2/M phases. Control, Ctrl.; Daunorubicin group (0.5 µM), DNR; liposomal daunorubicin (0.43 μm) group, Lip-DNR. Different letters above the columns indicate P < 0.05.

The results from assessing the impact of free and Lip-DNR on cell cycle stages by flow cytometry are presented in Figure 5A - C). As shown in Figure 5D, 11.7% of cells in the control group are in the G1 phase. Compared to the control group, the percentages of cells in the G1 phase for the DNR and Lip-DNR cell groups (0.43 μm) are 11.4% (P < 0.001) and 36.4% (P < 0.0001) respectively, indicating a significant increase. Additionally, the number of G1 phase cells in the Lip-DNR group is 22.2% higher than in the control group (P = 0.0001).

As per Figure 5E, the control group has 13.4% of cells in the S phase. The DNR and Lip-DNR groups show 12.8% (P = 0.013) and 23.4% (P = 0.0006) of cells in the S phase respectively, which is significantly higher than the control group. Additionally, the Lip-DNR group has 10.6% more S-phase cells than the DNR group (P = 0.03).

According to Figure 5F, 65.4% of cells in the control group were in the G2/M phase. However, both the DNR and Lip-DNR groups exhibited a significant decrease in the percentage of G2/M phase cells compared to the control group. Specifically, the DNR group had 41.8% (P < 0.0001) and the Lip-DNR group had 58% (P < 0.0001) of cells in the G2/M phase. Additionally, the percentage of G2/M phase cells in the Lip-DNR group was 15.2% lower than that in the DNR group (P = 0.0002).

After analyzing the cell stages in each group, it was observed that the control group predominantly comprised G2/S phase cells, indicating active proliferation (Figure 5G). The DNR group showed a more balanced distribution across the G1, S, and G2/M phases, with a reduction in G2/S phase cells and an increase in G1 and S phase cells compared to the control group, suggesting that DNR inhibits the proliferation of HCT116 cells. The Lip-DNR group had the lowest percentages of cells in the G2/S and G2/M phases compared to both the control group and the DNR group. Given that the concentration of Lip-DNR used (0.43 μm) closely matches that of free DNR (0.5 μm), this suggests that the liposomal form of DNR is more effective at inhibiting HCT116 cell proliferation than the free form.

## 5. Discussion

This study investigated the impact of Lip-DNR on inhibiting cell proliferation and inducing cell death in HCT116 cells via the PI3K/AKT/mTOR signaling pathway, comparing the effects of liposomal and free forms of daunorubicin. The liposomes containing daunorubicin were prepared using the thin layer hydration method. FE-SEM analysis confirmed that the liposomes are generally spherical and range from 50 to 100 nm in diameter, aligning with the size range for small unilamellar vesicles liposomes (SUVs, 30 - 100 nm) as reported by Fan (12).

Upon examining the morphology of HCT116 cells, it was observed that Lip-DNR induces apoptosis, or cell death. Research indicates that this effect results from the activation of caspase-9, ultimately leading to apoptosis (13). While the precise molecular mechanisms underpinning daunorubicin's antineoplastic properties remain elusive, it is suggested that oxidative stress induction, DNA intercalation, and inhibition of the topoisomerase II enzyme all contribute to its effectiveness (14).

Our study using the MTT method demonstrates an increase in the death rate of HCT116 cells with increasing concentrations of Lip-DNR. Furthermore, a concentration of 0.5 μm of DNR results in a higher number of dead cells compared to the control group. Several studies have shown that a 0.5 μm concentration of DNR induces apoptosis in various cells and cell lines (15-17). Additionally, we found that the cytotoxic effects of the liposomal form of DNR are dose-dependent, and this form is more effective at inducing cell death than the free form of daunorubicin. The induction of cell death following DNR treatment is attributed to an increase in intracellular ROS, which leads to DNA double-strand breaks (18). Moreover, treatment with DNR significantly increases the level of γH2AX in cells, indicating the induction of DNA double-strand breaks (19). When DNA double-strand breaks occur, they trigger the activation of PI3K-like kinases such as ataxia-telangiectasia mutated (ATM) (20). The activated ATM phosphorylates Ser139 residues at the carboxyl end of histone H2AX (H2AX), resulting in the formation of γH2AX around the DNA-DSB. This process leads to the accumulation of numerous γH2AX molecules around the DSB, which serve as binding sites for various DNA repair proteins and checkpoints, thus facilitating DNA-DSB repair (21). In response to DNA-DSB, ATM initiates the repair process through either non-homologous end joining (NHEJ) or homologous recombination (HR) (22).

Upon analyzing the expression of the PI3K gene, it was found that DNR can significantly reduce the transcription of PI3K in HCT116 cells. Moreover, the liposomal form of DNR demonstrated a greater effect on reducing PI3K gene transcription than the free form. PI3K expression plays a crucial role in various cellular functions, including cell proliferation, migration, glucose transport and catabolism, cytoskeleton rearrangement, and angiogenesis, which are vital in tumor initiation, progression, and maintenance (23). Numerous studies have shown that inhibiting PI3K leads to decreased cell proliferation and increased cell death (24). The efficacy of PI3K inhibitors in stopping tumor progression is well established (10). According to research by Lannutti et al., inhibiting PI3K activity reduces the phosphorylation of Akt and its downstream effectors, which increases the activity of Poly ADP-ribose polymerase, leading to the activation of caspase and induction of apoptosis (25). This research further underscores that PI3K inhibition reduces cell proliferation and increases cell death (24). Therefore, it can be concluded that treatment with DNR, in addition to enhancing cellular ROS, can induce apoptosis and cell death in the HCT116 cell line by reducing PI3K expression.

In this study, we explored the effect of DNR (both liposomal and free forms) on the cell cycle, demonstrating that treatment with DNR decreases the percentage of G2/M phase cells, indicative of inhibiting the proliferation of HCT116 cells. The inhibition of cell proliferation was more pronounced with the liposomal form than with the free form of DNR. Various factors influence the effectiveness of a chemotherapeutic agent, including concentration, duration of exposure, cell line doubling time, the state of DNA damage response mechanisms (DDR), and the type of damage incurred. The genetics of the cell line play a crucial role in determining how these factors impact effectiveness. DNR, like many other chemotherapeutic agents, disrupts cell proliferation by inhibiting DNA replication during the S phase of the cell cycle (19).

After inducing the toxic effects of daunorubicin, the cell first halts the cell cycle and then initiates DNA repair processes. Research by Al-Aamri et al. demonstrated that exposing SUP-B15 cells to DNR leads to a gradual accumulation of cells in the G1 phase. Similarly, treating MOLT-4 and CCRF-CEM cells with DNR results in these cells accumulating in the G2/M phase, with a corresponding decrease in the G1 and S phases (19). These findings align with previous studies on HL-60 cells (a myeloid leukemia cell line), where a 24-hour treatment with DNR caused an accumulation of cells at the G2/M checkpoint (26). A similar accumulation of CCRF-CEM cells in G2/M following treatment with the anthracycline doxorubicin has been observed (27). Doxorubicin also induced G2/M checkpoint arrest in HCT-116 human colon cancer cells, associated with p53 activation and the induction of p21 mRNA and protein expression (28). Regarding doxorubicin, studies have shown that its liposomal form possesses significantly better medicinal properties than the free drug (29).

### 5.1. Conclusions

The results of this study indicate that DNR inhibits the proliferation of HCT116 cells by reducing PI3K gene expression and increasing cell death. Furthermore, the liposomal form of this drug exhibits stronger effects than free daunorubicin.

## Data Availability

The dataset presented in the study is available on request from the corresponding author during submission or after publication.
